# Abdominal obesity and dsyglycemia are risk factors for liver fibrosis progression in NAFLD subjects: A population-based study

**DOI:** 10.3389/fendo.2022.1051958

**Published:** 2023-01-13

**Authors:** María Teresa Julián, Sílvia Ballesta, Guillem Pera, Alejandra Pérez-Montes de Oca, Berta Soldevila, Llorenç Caballería, Rosa Morillas, Carmen Expósito, Alba Martínez–Escudé, Manel Puig-Domingo, Josep Franch-Nadal, Pere Torán, Kenneth Cusi, Josep Julve, Dídac Mauricio, Núria Alonso

**Affiliations:** ^1^ Department of Endocrinology and Nutrition, Hospital Universitario Germans Trias I Pujol, Badalona, Barcelona, Spain; ^2^ Unitat de Suport a la Recerca (USR) Metropolitana Nord, Fundació Institut Universitari d’Investigació en Atenció Primària Jordi Gol i Gurina (IDIAP Jordi Gol), Mataró, Spain; ^3^ Department of Medicine, Universitat Autònoma de Barcelona, Barcelona, Spain; ^4^ Centro d’Investigaciones Biomédicas en Red, Enfermedades Hepatologia y Digestivas, Barcelona, Spain; ^5^ Hepatology Department, Hospital Germans Trias I Pujol, Badalona, Barcelona, Spain; ^6^ Primary Care, Centre d’Atenció Primària La Llagosta, Institut Català de la Salut, Barcelona, Spain; ^7^ Center for Biomedical Research on Diabetes and Associated Metabolic diseases (CIBERDEM), Instituto de Salud Carlos III, Madrid, Spain; ^8^ Primary Health Care Center Raval Sud, Gerència d’Atenció Primaria, Institut Català de la Salut, Barcelona, Spain; ^9^ Division of Endocrinology, Diabetes and Metabolism. University of Florida, Gainesville, FL, United States; ^10^ Department of Endocrinology and Nutrition, Hospital de la Santa Creu i Sant Pau; Institut d'Investigació Biomédica Sant Pau (IIB Sant Pau), Barcelona, Spain; ^11^ Department of Biochemistry and Molecular Biology, Universitat Autònoma de Barcelona, Barcelona, Spain; ^12^ Faculty of Medicine, University of Vic, Central University of Catalonia, Vic, Spain

**Keywords:** liver fibrosis, NAFLD, Transient elastography, abdominal obesity, dysglycemia, general population

## Abstract

**Objective:**

To investigate longitudinal changes in the liver stiffness measurement (LSM) in the general adult population without known liver disease and to describe its association with metabolic risk factors, with a special focus on subjects with non-alcoholic fatty liver disease (NAFLD) and dysglycemia.

**Material and Methods:**

A longitudinal adult population-based cohort study was conducted in Catalonia. LSM was measured by transient elastography (TE) at baseline and follow-up (median: 4.2 years). Subgroup with NAFLD and dysglycemia were analyzed. Moderate-to-advanced liver fibrosis was defined as LSM ≥8.0 kPa and LSM ≥9.2 kPa respectively.

**Results:**

Among 1.478 subjects evaluated, the cumulative incidence of LSM ≥8.0 kPa and ≥9.2 kPa at follow-up was 2.8% and 1.9%, respectively. This incidence was higher in NAFLD (7.1% for LSM ≥8.0 kPa and 5% for LSM ≥9.2 kPa) and dysglycemia (6.2% for LSM ≥8.0 kPa and 4.7% for LSM ≥9.2 kPa) subgroups. In the global cohort, the multivariate analyses showed that dysglycemia, abdominal obesity and atherogenic dyslipidemia were significantly associated with progression to moderate-to-advanced liver fibrosis. Female sex was negatively associated. In subjects with NAFLD, abdominal obesity and dysglycemia were associated with changes in LSM to ≥8.0 kPa and ≥9.2 kPa at follow-up. A decline in LSM value to <8 kPa was observed in 64% of those subjects with a baseline LSM ≥8.0 kPa.

**Conclusions:**

In this population study, the presence of abdominal obesity and dysglycemia were the main risk metabolic factors associated with moderate-to-advanced liver fibrosis development over time in general populations as well as in subjects with NAFLD.

## Introduction

Progressive liver fibrosis is the main determinant of long-term outcome in chronic liver diseases (CLDs). Although liver biopsy is considered the gold standard for assessing hepatic fibrosis, liver stiffness measurement (LSM) by transient elastography (TE) is one of the most widely used tools (1). LSM has been used to measure fibrosis in patients with various types of CLDs, including non-alcoholic fatty liver disease (NAFLD). However, few studies have investigated TE as a screening tool in the general population to identify asymptomatic subjects with liver fibrosis. Data from population-based studies revealed that 4-7% of subjects without known liver disease have significant liver fibrosis assessed by TE, mostly associated with NAFLD (2–6). In addition, several studies have evaluated the risk factors associated with significant liver fibrosis (2, 3). In a previous cross-sectional population-based study, we described that factors independently associated with increased LSM were male gender, abdominal obesity, type 2 diabetes (T2D) and dyslipidemia (6).

To date, we are not aware of studies that have evaluated longitudinal changes in LSM assessed by TE in the general population. Given that the natural history of CLD depends mainly on the underlying cause (7), most studies that evaluate the rate of fibrosis progression do it in a group of patients with the same CLD, with or without a specific treatment (7–9). The liver stiffness value by TE, as a non-invasive tool, may be useful for identifying progression to significant liver fibrosis over time in asymptomatic adult population.

The aim of the present study was to investigate the longitudinal changes of LSM assessed by TE and its associated metabolic risk factors within a well-characterized large Mediterranean population-based cohort without known liver disease. In addition, we also aimed to take the same approach in subjects with NAFLD and dysglycemia.

## Material and methods

### Study design and participants

This was a longitudinal population-based study based on a previous cross-sectional descriptive population study (5, 6). The study was conducted in several municipalities in the northern part of the Barcelona metropolitan area. The recruitment period was between April 2012 and January 2016, while a second cross-sectional evaluation was performed between October 2016 and December 2019. Participants in the study were randomly identified from a total of 162,950 subjects aged 18–75 years from the registries of the primary health care centers of the municipalities included in the study. Randomly-identified subjects were contacted through telephone and invited to participate. Patients with a current history of liver disease, including cholestasis, hepatitis C or B virus infection, and high-risk alcohol consumption were excluded from the study. Other exclusion criteria were active malignancy, other severe diseases (congestive heart failure New York Heart Association >2, chronic obstructive pulmonary disease defined as Global Initiative for Chronic Obstructive Lung Disease score >2, chronic kidney disease requiring dialysis, previous organ transplantation, and severe neurological diseases) or admission in long-term nursing homes. After obtaining informed consent, the following steps were taken: (i) detailed medical history, including alcohol consumption, anthropometric measurements, including body weight, height, body mass index (BMI), waist circumference, and arterial pressure. Abdominal obesity was described as waist circumference ≥102 cm in men or ≥88 cm in women; (ii) blood tests were performed after 12 hours of fasting, including liver biochemistry, hepatitis B and C virus markers (HBsAg and anti-HCV), serum fasting glycemia, glycosylated hemoglobin (HbA1c), serum creatinine, serum ferritin and serum lipid profile (total cholesterol (TC), LDL cholesterol (LDL-C), HDL cholesterol (HDL-C), non-HDL cholesterol (non-HDL-C), cholesterol remnants, triglycerides (TG) (iii) a TE with LSM was performed at baseline and at the end of the follow-up period.

### Definitions

The presence of NAFLD was defined using standard diagnostic criteria: one or more positive findings regarding fatty liver index (FLI ≥60), abdominal echography or liver biopsy. In our cohort, we excluded cases of viral hepatitis, high alcohol risk consumption and other liver disease. Thus, the evaluation of changes in LSM was to be primarily affected by metabolic disorders.

High-risk alcohol consumption was defined by standard drinking units/week; >21 in men and >14 in women. High serum transaminase concentrations were defined as aspartate transaminase (AST) and/or alanine transaminase (ALT) >40 IU/L. The FLI, FIB-4 index and NAFLD fibrosis score (NFS) was calculated as previously published (10–12). The FIB-4 index and NFS were considered as high risk of significant fibrosis according to cut-off points described in the original publications, being FIB-4 >2.67 and NFS ≥0.676 respectively (11, 12).

TE was performed using the Fibroscan^®^ system (402, Echosens^®^, Paris, France) by three trained specialist liver nurses. The XL probe was not available, so only the M probe was used. Moderate-to-advanced liver fibrosis was defined by LSM ≥8.0 kPa according to others epidemiological studies. Moreover, LSM ≥9.2 kPa was adopted to describe advanced fibrosis (2, 3, 13). This cut-off has been shown to predict significant liver fibrosis (F2-F4) with a high sensitivy and specificity in a previous large population-based study from Catalonian region (5) We defined progression or regression from one stage to another (< 8 a ≥8 kPa and < 9.2 to ≥ 9.2 and viceversa) but requiring, at least, a change (increase or decrease) of 1 kPa between LSM measurement at baseline and follow-up, based on previous studies (9, 14).

Type 2 diabetes mellitus and prediabetes diagnosis were based on a registered diagnosis in the clinical records or having an HbA1c ≥6.5% or fasting glucose ≥126 mg/dL and Hb1Ac 5.7-6.4% or fasting glucose 100-125 mg/dL, respectively. Abdominal obesity was define as waist circumference ≥102 cm in men or ≥88 cm in women. For analysis purposes, we included both T2D and prediabetes subjects under the predefined category of dysglycemia. Atherogenic dyslipidemia (AD) was defined as having >150 mg/dL TG and <40/50 (men/women) mg/dL HDL-C in serum. Remnant cholesterol concentration (expressed as mg/dL) was calculated using the following equation: total cholesterol – LCL-C – HDL-C.

### Statistical analysis

Descriptive analysis used frequencies and percentages (categorical variables), means and standard deviation (symmetric distributed continuous variables), and median and interquartile range (skewed continuous variables). Comparison of baseline vs follow-up means was assessed by paired t-Student tests and comparison of percentages by McNemar tests.

Univariate lineal regression was used to assess the association between different metabolic risk factors and progression of liver fibrosis (dependent variable). Progression was computed as the LSM at the end of follow-up minus LSM at baseline. Univariate logistic regression was performed to obtain the odds ratio (OR) and their 95% confidence intervals (95% CI) of these risk factors regarding the liver fibrosis progression, computed as changing, between the baseline and the end of follow-up, the LSM category from less than 8 to 8 or more kPa (or, less than 9.2 to 9.2 or more kPa). Different multivariate regression models, both of lineal and logistic analysis, were obtained adjusting for potential confounders. Only variables with a p value less than 0.1 in the univariate models were included in the adjusted models. The final multivariate models were selected only containing variables with p <0.05 and, if several models had different significant variables, the model with the best adjustment was selected. All comparisons were bilateral, and the significance was 0.05. Statistical analysis was performed using the Stata v17 statistical package.

### Ethics

The protocol was concordant with current and relevant guidelines and regulations and it was approved by the Ethics Committee of the Fundació Gol i Gorina (P11/58) (Barcelona, Spain). Written informed consent was obtained from all participants.

## Results

### Baseline characteristics of the study population

From a total of 4,866 invited subjects, 3,076 accepted participation in the study (participation rate, 63.2%). After exclusion of subjects with previous chronic liver disease (n = 13), high-risk alcohol consumption (n = 155), hepatitis B or C virus infection (n = 19) and not data available (n = 49), the final number at baseline was 2,840. Of these, 1,363 (48%) were not included in the second cross-sectional cut: 598 did not accept, 511 could not be located, 147 had moved from our area, and 107 were excluded for other reasons. Therefore, the final study population consisted of 1,478 subjects with 2 LSM, conducted at baseline and follow-up (median 4.2 years later; range, 3.0-5.5). Baseline and follow-up clinical and biochemical characteristics of this cohort are shown in **Table 1**. The prevalence of moderate-to-advanced liver fibrosis by TE (LSM ≥8.0 kPa and ≥9.2 kPa cut-off) was 4.7% (n = 70) and 2.5% (n = 37), respectively. Comparison between subjects who completed and not the follow-up are represented in **Supplementary Table 1**.

**Table 1:** Characteristics of the study population at baseline and follow-up.

	Total (n = 1.478)			NAFLD (n = 511)			Dysglycemia (n = 365)		
	Baseline	Follow-up	p	Baseline	Follow-up	p	Baseline	Follow-up	p
Age, years	56 ± 11	60 ± 11	–	59 ± 9	63 ± 9	–	61 ± 8	65 ± 8	–
Female, n (%)	918 (62)	918 (62)	–	253 (50)	253 (50)	–	190 (52)	190 (52)	–
Body mass index ≥ 30, kg/m2	28 ± 5	28 ± 5	<0.001	32 ± 4	32 ± 4	0.165	30 ± 5	30 ± 5	0.097
Abdominal obesity, n (%)	743 (51)	805 (55)	<0.001	422 (83)	402 (79)	0.010	231 (64)	247 (69)	0.036
Dysglycemia, n (%)	365 (25)	393 (27)	0.044	214 (42)	230 (45)	0.095	365 (100)	282 (77)	<0.001
Glucose, mg/dL	100 ± 24	100 ± 23	0.082	108 ± 31	110 ± 29	0.285	125 ± 35	122 ± 32	0.146
Glycated hemoglobin, %	5.7 ± 0.7	5.7 ± 0.7	0.004	5.9 ± 0.9	6.0 ± 0.9	0.042	6.4 ± 1.0	6.4 ± 1.0	0.522
Triglycerides, mg/dL	119 ± 71	115 ± 57	0.0121	161 ± 92	142 ± 65	<0.001	154 ± 96	140 ± 68	0.002
Total cholesterol, mg/dL	14 ± 38	208 ± 39	<0.001	216 ± 40	203 ± 44	<0.001	208 ± 39	197 ± 45	<0.001
LDL cholesterol, mg/dL	135 ± 33	130 ± 34	<0.001	135 ± 36	124 ± 38	<0.001	127 ± 35	118 ± 39	<0.001
HDL cholesterol, mg/dL	56 ± 13	55 ± 13	0.300	51 ± 11	50 ± 11	0.084	51 ± 12	51 ± 12	0.937
Cholesterol remnants†, mg/dL	23 ± 13	23 ± 14	0.517	30 ± 15	29 ± 18	0.071	29 ± 16	29 ± 21	0.924
Atherogenic dyslipidemia§, n (%)	123 (9)	137 (10)	0.237	90 (19)	89 (18)	0.910	66 (19)	71 (20)	0.522
ALT and/or AST >40 U/L, n (%)	105 (8)	102 (7)	0.798	69 (14)	56 (12)	0.128	43 (12)	29 (8)	0.035
FLI¶	47 ± 28	49 ± 28	0.001	79 ± 13	74 ± 19	<0.001	65 ± 26	63 ± 26	0.147
FLI ≥60, n (%)	450 (35)	481 (37)	0.032	450 (96)	364 (78)	<0.001	198 (59)	195 (58)	0.696
NAFLD ||, n (%)	484 (36)	677 (50)	<0.001	484 (100)	415 (86)	<0.001	205 (59)	245 (71)	<0.001
Mean liver fibrosis by LSM (kPa)	4.8 ± 2.2	4.9 ± 2.0	0.048	5.8 ± 3.0	5.8 ± 2.8	0.842	5.8 ± 3.4	5.8 ± 3.1	0.961
Liver fibrosis by LSM ≥8.0 kPa, n (%)	70 (5)	63 (4)	0.448	62 (12)	54 (11)	0.346	43 (12)	38 (10)	0.456
Liver fibrosis by LSM ≥9.2 kPa, n (%)	37 (3)	41 (3)	0.572	34 (7)	37 (7)	0.655	26 (7)	28 (8)	0.715
FIB-4 >2.67, n (%)	22 (2)	51 (4)	<0.001	11 (2)	24 (5)	0.005	6 (2)	15 (5)	0.007
High NFS, n (%)	15 (1)	73 (6)	<0.001	11 (2)	55 (12)	<0.001	14 (4)	48 (15)	<0.001

Data are n (%) or mean ± SD.

ALT, alanine aminotransferase; AST, aspartate aminotransferase; FLI, fatty liver index; GGT, gamma-glutamyltransferase; HDL, high-density lipoprotein; LDL, low-density lipoprotein; LSM, liver stiffness measurement (by elastography); NAFLD, non-alcoholic fatty liver disease.

^†^Cholesterol remnants: total cholesterol- LDL-C-HDL-C.

^‡^Non-HDL cholesterol is defined as the difference between total and HDL-C.

^§^Atherogenic dyslipidemia is defined by triglyceride >150 mg/dL and HDL-C <40 mg/dL in men and <50 mg/dL in women.

^¶^FLI (fatty liver index) estimates the amount of fat in the liver and includes body mass index, waist circumference, and serum gamma-glutamyltransferase and triglycerides.

^||^NAFLD (nonalcoholic fatty liver disease) was defined using standard diagnostic criteria (FLI, abdominal echography or liver biopsy. - Not included in the model.

### Longitudinal changes in LSM and factors associated within the whole study group

**Figure 1:**
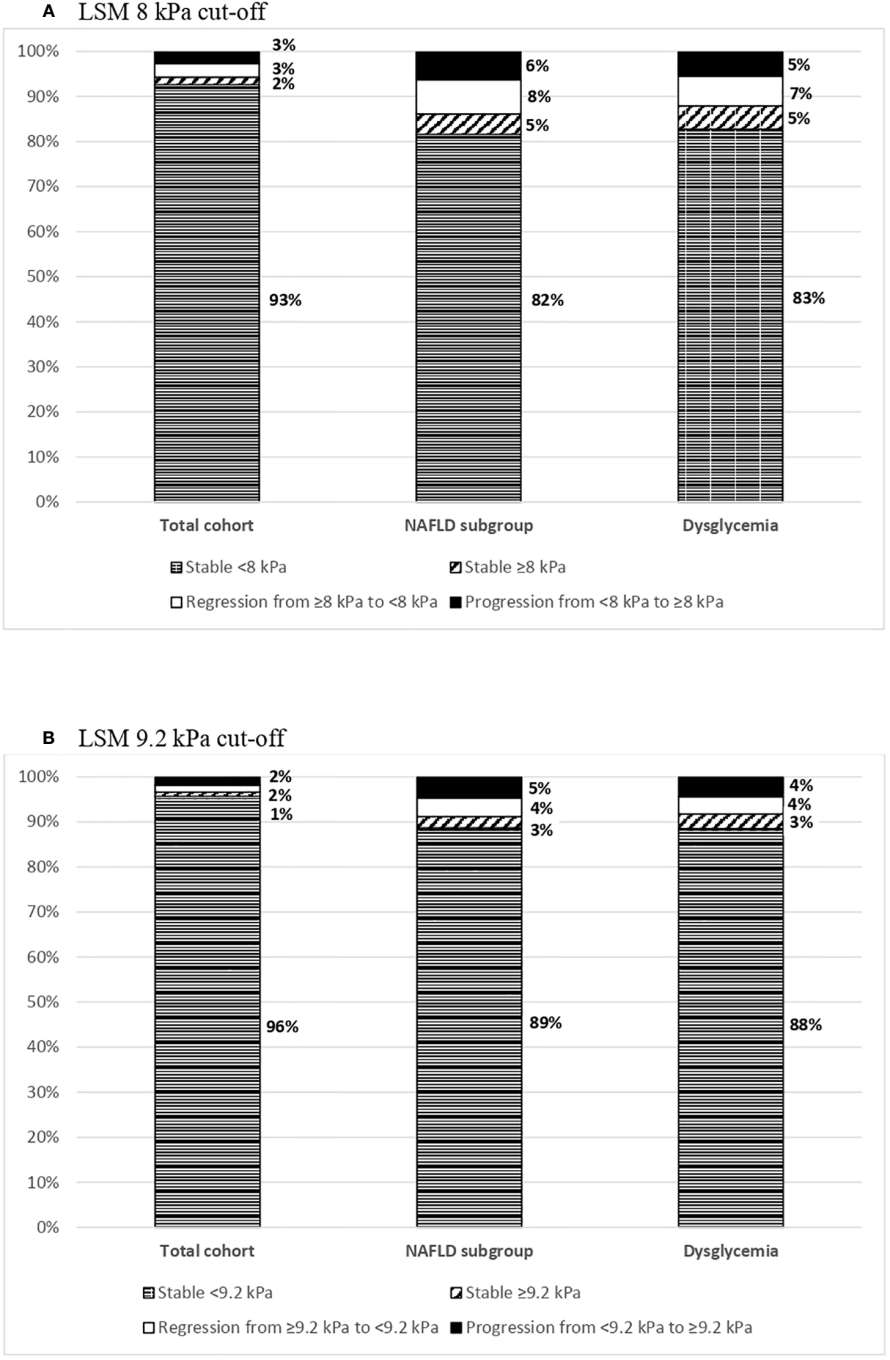
Categorical changes in longitudinal LSM by stage at follow up. **(A)** Percentage of subjects who maintained stable, regressed or progressed LSM during the follow-up period for the LSM 8 kPa cut-off. The total cohort, the NAFLD subgroup and the dysglycemia subgroup are represented in bars. To consider a change, a minimum increase/decrease of 1 kPa was required. **(B)** Percentage of subjects who maintained stable, regressed or progressed LSM during the follow-up period for the LSM 9.2 kPa cut-off. The total cohort, the NAFLD subgroup and the dysglycemia subgroup are represented in bars. To consider a change, a minimum increase/decrease of 1 kPa was required.

Categorical changes in longitudinal LSM according to the stage of baseline TE (<8 kPa or ≥ 8 kPa and < 9.2 or ≥9.2 kPa) are represented in **Figure 1**. The cumulative incidence for liver fibrosis progression in the whole follow-up was 2.8% for LSM ≥8.0 kPa and 1.9% for LSM ≥9.2 kPa. On the other hand, a decline in LSM to < 8 kPa was observed in 46 (64%) subjects with a baseline LSM ≥8.0 at follow-up. The LSM value decreased from ≥9.2 kPa at baseline to <9.2 kPa over time in 23 (62%) subjects. Moreover, 19 (51%) subjects experienced a LSM decrease from ≥9.2 kPa to < 8 kPa (**Supplementary Table 2A**).

Subsequently, the risk factors associated with progression in mean LSM were analyzed. In the univariate lineal regression analysis, factors associated with a higher increase in the mean LSM value at follow-up were dysglycemia, obesity and abdominal obesity (**Supplementary Table 3A**). After adjustment, the presence of abdominal obesity and dysglycemia were the two main conditions associated with significant increase in mean LSM during the follow-up period (**Table 2A**). Female sex was associated with a higher decline in the mean LSM value over time.

**Table 2:** Multivariate analysis of risk factors associated with an increase in LSM over time.

a) Total cohort				
	beta	95% CI		P value
Dysglycemia	0.27	0.09	0.46	0.004
Abdominal obesity	0.27	0.11	0.43	0.001
Female sex	-0.34	-0.50	-0.17	<0.001
b) NAFLD subgroup				
	beta	95% CI		P value
Dysglycemia *	0.49	0.13	0.85	0.008
c) Dysglycemia subgroup				
	beta	95% CI		P value
Abdominal obesity	0.46	0.02	0.90	0.039

CI, confidence interval; LSM, liver stiffness measurement (by elastography).

Variables in each model mutually adjusted.

*This is the only variable that is significant (p < 0.05), so it is a univariate model.

Next, we investigated the factors positively associated with progression to moderate-to-advanced liver fibrosis. In the univariate logistic regression model, the metabolic factors positively associated were: dysglycemia, obesity, abdominal obesity, AD, cholesterol remnant and HDL-C (**Supplementary Table 4A**). After multivariate analyses, dysglycemia, abdominal obesity and AD, were significantly associated with LSM progression for both cut-offs (≥8.0 kPa and ≥9.2 kPa), whereas female sex (ORs 0.3) was negatively associated (**Table 3A**).

**Table 3:** Multivariate logistic regression analysis of risk factors associated with LSM ≥ 8 kPa and ≥ 9.2 kPa at follow-up.

a) Total cohort								
	*LSM ≥ 8 kPa*				*LSM ≥ 9.2 kPa*			
	OR	95% CI		P value	OR	95% CI		P value
Dysglycemia	2.4	1.2	4.7	0.013	3.1	1.4	7.3	0.008
Abdominal obesity	6.8	2.7	16.9	0.000	5.5	1.8	16.7	0.003
Female sex	0.3	0.1	0.5	0.000	0.3	0.2	0.8	0.012
Atherogenic dyslipidemia	2.4	1	5.4	0.040	2.8	1.1	6.9	0.026
b) NAFLD subgroup								
	*LSM ≥ 8 kPa*				*LSM ≥ 9.2 kPa*			
	OR	95% CI		P value	OR	95% CI		P value
Dyglycemia	2.4	1.1	5	0.020	3.2	1.3	7.6	0.001
Abdominal obesity	5	1.1	22	0.034	–	–	–	NS
Female sex	0.5	0.2	0.9998	0,05	–	–	–	NS
Body mass index ≥ 30	–	–	–	NS	5.3	1.2	22.7	0.026
c) Dysglycemia subgroup								
	*LSM ≥ 8 kPa*				*LSM ≥ 9.2 kPa*			
	OR	95% CI		P value	OR	95% CI		P value
Abdominal obesity	10.5	2.3	47.4	0.002	9.8	1.3	75.3	0.028
Female sex	0.2	0.1	0.6	0.002	–	–	–	NS

CI, confidence interval; LSM: liver stiffness measurement (by elastography).

NS: p > 0.05. Not included in the model.

Variables in each model mutually adjusted.

Mean changes in metabolic parameters (body weight, waist circumference, plasma glucose concentrations and HbA1c) from baseline according to progression/regression in liver fibrosis at follow-up are represented in **Supplementary Table 5**. Interestingly, the mean weight gain among subjects that experimented progression to moderate-to-advanced liver fibrosis (LSM < 8.0 to ≥ 8 kPa or LSM <9.2 to ≥9.2 kPa), was 3.2 and 3.5 Kg, respectively. On the other hand, an average weight loss of -2.5 and -5.3 kg was observed in those subjects with decreased in LSM to < 8 kPa and < 9.2 kPa. Changes were also observed in the evolution of waist circumference, plasma glucose and HbA1c between those subjects who either showed a progression/regression in LSM during follow-up.

### Longitudinal changes in LSM and associated factors in NAFLD subjects

Among the entire cohort, 511 (36%) subjects met the criteria for NAFLD. Baseline and follow-up clinical and biochemical characteristics are shown in **Table 1**. Changes in longitudinal LSM according to the stage of baseline TE (<8 kPa or ≥ 8 kPa and < 9.2 or ≥9.2 kPa) are represented in **Figure 1**. The cumulative incidence for liver fibrosis progression was 7.1% for LSM ≥8.0 kPa and 5.0% for LSM ≥9.2 kPa. At follow-up, a decline in LSM value to < 8 kPa was observed in 39 (63%) subjects with baseline LSM ≥8.0 kPa. The LSM value decreased from ≥9.2 kPa at baseline to <9.2 kPa in 62% (n=21/34) and to <8 kPa in 17 (50%) subjects over time (**Supplementary Table 2B**).

In multivariate lineal regression, the presence of dysglycemia was associated with a significant increase in the mean LSM value follow-up time (**Table 2B**). Factors independently associated with LSM progression to ≥8.0 kPa in the multivariate logistic regression analysis were dysglycemia and abdominal obesity (**Table 3B**). When a cut-off of LSM ≥9.2 kPa was analyzed, dysglycemia and obesity were significantly associated with LSM progression. Female sex was negatively associated with LSM progression to ≥8.0 kPa, but not with LSM ≥9.2 kPa.

### Longitudinal changes in LSM and associated factors in subjects with dysglycemia

Next, because we found that dysglycemia is one of the main factors associated with liver fibrosis progression, we analyzed the same endpoints in subjects with impaired glucose metabolism. Among the entire cohort, 365 (25%) subjects had dysglycemia. Baseline clinical and biochemical characteristics are shown in **Table 1**. Changes in longitudinal LSM according to the stage of baseline TE (<8 kPa or ≥ 8 kPa and < 9.2 or ≥9.2 kPa) are represented in **Figure 1**. The cumulative incidence was 6.2% for LSM ≥8.0 kPa and 4.7% for LSM ≥9.2 kPa. At follow-up, a decline in LSM value to <8 kPa was observed in 24 (56%) subjects with a baseline LSM ≥8.0 kPa (n = 31), and in 14 (54%) subjects with a baseline LSM ≥9.2 kPa (**Supplementary Table 2C**).

After adjustment, the presence of abdominal obesity was the only factor associated with higher increase in the mean LSM value (**Table 2C**) and with an increase in LSM to ≥8.0 kPa and ≥9.2 kPa over time in a multivariate logistic regression analysis (**Table 3C**). Female sex was negatively associated with an increased in LSM but only for ≥8.0 kPa cut-off.

## Discussion

To our knowledge, this is the first longitudinal study aimed at investigating changes of LSM over time assessed by TE in a large well-characterized population-based cohort, with special attention in analyzing those risks factors associated with progression to moderate-to-advanced liver fibrosis. Of note, we excluded secondary causes of liver disease, and thus the cause of liver fibrosis was primarily NAFLD. Several community-based studies, including previous data from our current cohort (5, 6), have reported that the prevalence of liver fibrosis obtained by TE is high (ranging between 5.6% and 7.5%), mostly associated to NAFLD. This prevalence is higher among individuals with associated metabolic risk factors, such as diabetes (18%-27%) (6, 15–20).

In our cohort, using a cut-off value of LSM ≥8.0 kPa and ≥9.2 kPa by TE, the cumulative incidence of moderate-to-advanced liver fibrosis was 2.8% and 1.9%, respectively, over a median period of around 4 years. Interestingly, extrapolation of our data to the European population between 18-75 years (312 million in 2018) indicates that approximately 8.7 million European citizens with unknown liver disease could develop significant liver fibrosis in a period around 4 years. In addition, as expected, when we focus on the subgroup of subjects with NAFLD or dysglycemia, the incidence of significant liver fibrosis at follow up was higher than in the general population. In a previous cross-sectional population study, we recently published that the prevalence of liver fibrosis assessed by TE was higher in subjects with NAFLD (13% vs 1%) and T2D (21% vs 3.4%) compared with those without these metabolic conditions (6).

Further, we analyzed the metabolic risk factors associated with progression to moderate-to-advanced liver fibrosis during follow-up period. In the current study, we found that abdominal obesity and dysglycemia are the main risk factors independently associated with longitudinal LSM changes and progression to moderate-to advanced liver fibrosis in the whole cohort. These risk factors also have been described in cross-sectional studies as factors independently associated with increased LSM in the general population, apart from serum glucose, HDL-C, and TG levels (5). We also observed that the presence of abdominal obesity increase the risk for liver fibrosis progression in all groups analyzed. Some studies have examined the longitudinal changes of LSM in NAFLD from outpatient clinic, not in the general population (8, 14). In NAFLD subjects (n = 507), Mikolasevic et al. described a progression rate to significant fibrosis assessed by TE (LSM ≥7 kPa) of 17.7% during a median follow-up period of 1.8 years. In that study, obese patients had the highest risk of progression (14). Lallukka et al. described that 29% of NAFLD subjects (n = 97) had increased LSM after a median follow-up period of 11.3 years, with baseline liver fat (measured by proton magnetic resonance spectroscopy) being an independent predictor of increased LSM (8). We must highlight that, due to the correlation between the potential risk factors associated with an LSM increase, only a few factors achieved statistical significance. This was more evident in models where abdominal obesity was found to be an independent factor for the increase of LSM. Both risk factors, BMI and abdominal obesity, could not be included in the model due to high collinearity. The model with the best statistical adjustment was selected, but this does not mean that the excluded variable had no effect.

Regarding the lipid profile, AD was a factor independently associated with progression to moderate-to-advanced liver fibrosis in the whole population. High levels of TG and/or low HDL-C have been reported to be associated with higher LSM in some other population-based studies (5, 21). In our previous report, AD dyslipidemia was independently associated with moderate-to-advanced liver fibrosis in subjects with T2D and NAFLD but not in those without this condition. Furthermore, we also found female sex as a protective factor against the development liver fibrosis in general cohort. This sexual dimorphism in liver fibrosis risk has also been previously described for other cardiovascular risk factors, and one hypothesis is that a protective role of estrogens may exist (22).

Highlight in our study, we found that 64% of subjects with LSM ≥8.0 kPa at baseline showed a decline <8 kPa at follow-up. Similarly, results occur in NAFLD and dysglycemia subgroups. Of note, in some liver conditions, such as alcoholic liver disease or viral hepatic infection, effective treatment can change the natural history of underlying CLD, resulting in a regression and/or lower progression rate to liver fibrosis (23). Although no specific pharmacological treatment has yet been established for fatty liver disease (23), it is known that a weight loss > 7% can induce liver fibrosis regression (24). If we assume that in our cohort the cause of liver fibrosis was primarily NAFLD, the optimization of the associated metabolic risk factors, including glycemic control and body weight loss, might have contributed, at least in part, to the decline in LSM. In this sense, among subjects that changed from one stage to another (LSM ≥ 8.0 to <8 kPa or LSM ≥9.2 to <9.2 kPa), we observed a decreased in body weight, waist circumference and parameters of glycemic control during the follow-up period No specific pharmacological or nutritional intervention was performed during the study period. The usual clinical practice was followed.

The major strengths of the current investigation are as follows: (i) this is a population-based study; (ii) the prospective design; (iii) the large sample size analyzed and (iv) the measurement of LSM in all participants was performed by the same experienced operator to further improve the accuracy and reduce the variability of TE measurements.

However, our study had some limitations that deserve mentioning: (i) Data from the present study revealed that changes in LSM values were relatively small during the follow-up period in the whole population. This finding might be explained by several reasons. First, the study was conducted in a community setting and not in hospital-based clinics or reference Hepatology units, where subjects are usually referred for known liver disease. Second, we excluded subjects with previous liver disease, including hepatitis viral infection or high-risk alcohol consumption. Third, it is probable that a longer follow-up time is needed to identify changes in LSM in a general population; (ii) the XL probe was not available in this study, but the percentage of unreliable measurements of liver stiffness was only 1.5% as our population was not overtly obese; actually, few patients had class 2 and 3 obesity. The use of an XL probe could reduce this failure rate, but we do not believe it would have impacted our main results”; (iii) finally, data on drug treatment of the study subjects was not available.

## Conclusions

We hereby report for the first time that minimal changes in LSM values occurred in a Mediterranean population without known liver disease over a median of 4.2 years follow-up time. In a general population, metabolic risk factors such as abdominal obesity, dysglycemia and atherogenic dyslipidemia are associated with a risk of progression to moderate-to-advanced liver. Specifically, abdominal obesity is a common risk factor for liver fibrosis progression in all groups evaluated. Of note, in a non-negligible percentage of subjects in our cohort, a decline in LSM was observed. Weight loss and control of other associated metabolic factors may have contributed to the regression of liver fibrosis at follow-up.

## Data availability statement

The raw data supporting the conclusions of this article will be made available by the authors, without undue reservation.

## Ethics statement

The studies involving human participants were reviewed and approved by Ethics Committee of the Fundació Gol i Gorina (P11/58) (Barcelona, Spain). The patients/participants provided their written informed consent to participate in this study.

## Author contributions

MJ and SB interpreted the data and wrote the manuscript. NA, GP, LC and DM designed the study, interpreted data, and reviewed the manuscript. AP-M, BS, RM, CE, MP-D, JF-N, PT, KC, JJ contributed to reviewed the manuscript. All authors contributed significantly to the work and approved the final article. All authors contributed to the article and approved the submitted version.
