# Supplementary material for: Abdominal obesity and dsyglycemia are risk factors for liver fibrosis progression in NAFLD subjects: A population-based study
Source: Front Endocrinol (Lausanne). 2023 Jan 13;13:1051958. doi: 10.3389/fendo.2022.1051958 (PMC9880071; doi:10.3389/fendo.2022.1051958)
Supplement: Supplementary file 1 [file DataSheet_1.docx]

SUPPLEMENTARY FIGURE 1. Flow chart of the study

Exclusions due to known chronic liver disease n = 2004 (1.23%)

Adult population between 18-75 years: 162950 citizens

Target population: 160946 citizens

No acceptance n = 1790 (36.8%)

Excluded subjects: previous chronic liver disease (n = 13), high-risk alcohol consumption (n = 155), hepatitis B or C virus infection (n = 19), not data available (n = 49)

- c

c

Population invited to participate (random): 4866 subjects

Sample analyzed: 1478 subjects

- c

Excluded subjects in the second cross-sectional cut: not accept (n = 598), not be located (n = 511), moved from our area (n = 147), other reasons (n = 107)

Sample included: 2840 subjects

Population accepted to participated: 3076

(participation rate, 63.2%)

SUPPLEMENTARY TABLE 1. Comparison between subjects who completed and not the follow-up period

|  | DROP OUT (n=1233) | FOLLOW-UP (n=1478) |  |  |  |
| --- | --- | --- | --- | --- | --- |
|  | n/mean | sd/% | n/mean | sd/% | p |
| Age, years | 52 | ±13 | 56 | ±11 | 0.000 |
| Female, n (%) | 729 | (59%) | 918 | (62%) | 0.108 |
| Body mass index, *kg/m^2^* | 28 | ±5 | 28 | ±5 | 0.864 |
| Abdominal obesity, n (%) | 597 | (49%) | 751 | (51%) | 0.273 |
| Dysglycemia, n (%) | 289 | (23%) | 365 | (25%) | 0.465 |
| Glucose, *mg/dL* | 101 | ±27 | 99 | ±24 | 0.058 |
| Glycated hemoglobin (%) | 5,7 | ±0,7 | 5,7 | ±0,7 | 0.917 |
| Triglyceride, *mg/dL* | 123 | ±78 | 120 | ±72 | 0.240 |
| Total cholesterol, *mg/dL* | 210 | ±40 | 214 | ±38 | 0.010 |
| LDL-cholesterol, *mg/dL* | 132 | ±36 | 135 | ±33 | 0.024 |
| HDL-cholesterol, *mg/dL* | 54 | ±12 | 56 | ±13 | 0.015 |
| Atherogenic dyslipemia^§^, n (%) | 128 | (11%) | 135 | (9%) | 0.150 |
| ALT and/or AST > 40 U/L, n (%) | 101 | (9%) | 111 | (8%) | 0.367 |
| FLI^¶^ | 47 | ±30 | 47 | ±28 | 0.950 |
| Mean liver fibrosis by LSM (kPa) | 5,0 | 1,9 | 4,8 | 2,2 | 0.119 |
| FIB-4 >2.67, n (%) | 18 | (2%) | 24 | (2%) | 0.846 |
| High NFS, n (%) | 28 | (3%) | 17 | (1%) | 0.009 |

SUPPLEMENTARY TABLE 2. Changes in longitudinal LSM by stage at follow up

1. Total cohort

|  | **Follow-up** | |
| --- | --- | --- |
| **Baseline** | **<8 kPa** | **≥8 kPa** |
| **<8 kPa**  (n = 1408)(n/%) | 1369 (97) | 39 (3) |
| **≥8 kPa**  (n = 70) (n/%) | 45 (64) | 25 (36) |

|  | **Follow-up** | |
| --- | --- | --- |
| **Baseline** | **<9.2 kPa** | **≥9.2 kPa** |
| **<9.2 kPa**  (n = 1441)(n/%) | 1414 (98) | 27 (2) |
| **≥9.2 kPa**  (n = 37) (n/%) | 23 (62) | 14 (38) |

1. NAFLD subgroup

|  | **Follow-up** | |
| --- | --- | --- |
| **Baseline** | **<8 kPa** | **≥8 kPa** |
| **<8 kPa**  (n = 449)(n/%) | 417 (93) | 32 (7) |
| **≥8 kPa**  (n = 62) (n/%) | 39 (63) | 23 (37) |

|  | **Follow-up** | |
| --- | --- | --- |
| **Baseline** | **<9.2 kPa** | **≥9.2 kPa** |
| **<9.2 kPa**  (n =477)(n/%) | 453 (95) | 24 (5) |
| **≥9.2 kPa**  (n = 34) (n/%) | 21 (62) | 13 (38) |

1. Dysglycemia subgroup

|  | **Follow-up** | |
| --- | --- | --- |
| **Baseline** | **<8 kPa** | **≥8 kPa** |
| **<8 kPa**  (n = 322)(n/%) | 302 (94) | 20 (6) |
| **≥8 kPa**  (n = 43) (n/%) | 24 (56) | 19 (44) |

|  | **Follow-up** | |
| --- | --- | --- |
| **Baseline** | **<9.2 kPa** | **≥9.2 kPa** |
| **<9.2 kPa**  (n = 339)(n/%) | 323 (95) | 16 (5) |
| **≥9.2 kPa**  (n = 26) (n/%) | 14 (54) | 12 (46) |

* To consider a change, a minimum increase/decrease of 1 kPa was required.

SUPPLEMENTARY TABLE 3. Univariate lineal regression analysis of risk factors associated with increased in LSM over time.

1. Total cohort

|  | beta | | 95% CI | | | | P value | |
| --- | --- | --- | --- | --- | --- | --- | --- | --- |
| Dysglycemia | 0.34 | | 0.16 | | 0.52 | | <0.001 | |
| Hypertension | 0.16 | | -0.01 | | 0.33 | | 0.072 | |
| Body mass index ≥ 30 | 0.33 | | 0.16 | | 0.50 | | <0.001 | |
| Abdominal obesity | 0.21 | 0.06 | | 0.37 | | 0.007 | |  |
| Atherogenic dyslipemia† | 0.25 | | -0.02 | | 0.52 | | 0.068 | |
| Cholesterol remnants | 0.002 | | -0.004 | | 0.01 | | 0.562 | |
| LDL cholesterol | -0.0005 | | -0.003 | | 0.002 | | 0.692 | |
| HDL cholesterol < 40/50 | 0.13 | | -0.07 | | 0.32 | | 0.200 | |
| Age | -0.003 | | -0.01 | | 0.004 | | 0.383 | |
| Female sex | -0.28 | | -0.44 | | -0.13 | | <0.001 | |

1. NAFLD subgroup

|  | beta | | 95% CI | | | | P value | |
| --- | --- | --- | --- | --- | --- | --- | --- | --- |
| Dysglycemia | 0.49 | | 0.13 | | 0.85 | | 0.008 | |
| Hypertension | 0.15 | | -0.20 | | 0.51 | | 0.406 | |
| Body mass index ≥ 30 | 0.11 | | -0.27 | | 0.50 | | 0.555 | |
| Abdominal obesity | 0.20 | -0.27 | | 0.66 | | 0.413 | |  |
| Atherogenic dyslipemia† | 0.29 | | -0.15 | | 0.74 | | 0.199 | |
| Cholesterol remnants | -0.01 | | -0.02 | | 0.01 | | 0.289 | |
| LDL cholesterol | -0.001 | | -0.01 | | 0.004 | | 0.775 | |
| HDL cholesterol < 40/50 | 0.21 | | -0.17 | | 0.60 | | 0.275 | |
| Age | -0.01 | | -0.03 | | 0.01 | | 0.362 | |
| Female sex | -0.22 | | -0.57 | | 0.13 | | 0.219 | |

1. Dysglycemia subgroup

|  | beta | 95% CI | | P value |
| --- | --- | --- | --- | --- |
| Hypertension | -0.05 | -0.47 | 0.37 | 0.807 |
| Body mass index ≥ 30 | 0.47 | 0.05 | 0.90 | 0.028 |
| Abdominal obesity | 0.46 | 0.02 | 0.90 | 0.039 |
| Atherogenic dyslipemia† | -0.12 | -0.66 | 0.43 | 0.676 |
| Cholesterol remnants | -0.01 | -0.02 | 0.01 | 0.087 |
| LDL cholesterol | 0.0005 | -0.01 | 0.01 | 0.880 |
| HDL cholesterol < 40/50 | 0.20 | -0.26 | 0.65 | 0.394 |
| Age | -0.02 | -0.05 | 0.01 | 0.137 |
| Female sex | -0.15 | -0.56 | 0.27 | 0.480 |

†Atherogenic dyslipidemia is defined by triglyceride > 150 mg/dL and HDL-C < 40 mg/dL in men and < 50 mg/dL in women.

SUPPLEMENTARY TABLE 4. Univariate logistic regression analysis of risk factors associated with LSM ≥ 8 kPa and ≥ 9.2 kPa at follow-up.

1. Total cohort

|  | *LSM ≥ 8 kPa* | | | | *LSM ≥ 9.2 kPa* | | | |
| --- | --- | --- | --- | --- | --- | --- | --- | --- |
|  | OR | 95% CI | | P value | OR | 95% CI | | P value |
| Dysglycemia | 3.7 | 2.0 | 7.1 | 0.000 | 4.9 | 2.3 | 10.7 | 0.000 |
| Hypertension | 1.7 | 0.9 | 3.2 | 0.138 | 2.7 | 1.3 | 5.8 | 0.011 |
| Body mass index ≥ 30 | 5.4 | 2.7 | 10.6 | 0.000 | 14.7 | 5.0 | 42.8 | 0.000 |
| Abdominal obesity | 5.8 | 2.4 | 14.0 | 0.000 | 5.8 | 2.0 | 17.0 | 0.001 |
| Atherogenic dyslipemia† | 3.7 | 1.7 | 8.0 | 0.001 | 4.9 | 2.1 | 11.5 | 0.000 |
| Cholesterol remnants | 1.0 | 1.0 | 1.0 | 0.003 | 1.0 | 1.0 | 1.0 | 0.029 |
| LDL cholesterol | 1.0 | 1.0 | 1.0 | 0.545 | 1.0 | 1.0 | 1.0 | 0.502 |
| HDL cholesterol < 40/50 | 2.1 | 1.0 | 4.2 | 0.041 | 2.7 | 1.2 | 6.0 | 0.015 |
| Age | 1.0 | 1.0 | 1.0 | 0.750 | 1.0 | 1.0 | 1.1 | 0.244 |
| Female sex | 0.4 | 0.2 | 0.8 | 0.005 | 0.5 | 0.2 | 1.0 | 0.055 |

1. NAFLD subgroup

|  | *LSM ≥ 8 kPa* | | | | *LSM ≥ 9.2 kPa* | | | |
| --- | --- | --- | --- | --- | --- | --- | --- | --- |
|  | OR | 95% CI | | P value | OR | 95% CI | | P value |
| Dysglycemia | 2.5 | 1.2 | 5.2 | 0.014 | 3.2 | 1.4 | 7.7 | 0.008 |
| Hypertension | 1.02 | 0.5 | 2.1 | 0.949 | 1.5 | 0.7 | 3.4 | 0.342 |
| Body mass index ≥ 30 | 1.8 | 0.8 | 4.3 | 0.180 | 5.4 | 1.3 | 23.2 | 0.024 |
| Abdominal obesity | 3.5 | 0.8 | 14.7 | 0.095 | 2.4 | 0.6 | 10.4 | 0.243 |
| Atherogenic dyslipemia† | 2.0 | 0.9 | 4.4 | 0.101 | 2.4 | 0.98 | 5.7 | 0.056 |
| Cholesterol remnants | 1.003 | 0.98 | 1.03 | 0.790 | 0.996 | 0.97 | 1.03 | 0.798 |
| LDL cholesterol | 1.003 | 0.99 | 1.01 | 0.595 | 0.995 | 0.98 | 1.01 | 0.424 |
| HDL cholesterol < 40/50 | 1.6 | 0.7 | 3.3 | 0.233 | 1.8 | 0.8 | 4.2 | 0.156 |
| Age | 0.99 | 0.95 | 1.03 | 0.768 | 1.01 | 0.97 | 1.1 | 0.567 |
| Female sex | 0.6 | 0.3 | 1.2 | 0.136 | 0.7 | 0.3 | 1.6 | 0.410 |

1. Dysglycemia subgroup

|  | *LSM ≥ 8 kPa* | | | | *LSM ≥ 9.2 kPa* | | | |
| --- | --- | --- | --- | --- | --- | --- | --- | --- |
|  | OR | 95% CI | | P value | OR | 95% CI | | P value |
| Hypertension | 0.9 | 0.3 | 2.2 | 0.768 | 1.6 | 0.6 | 4.5 | 0.339 |
| Body mass index ≥ 30 | 5.9 | 1.9 | 18.1 | 0.002 | 20.6 | 2.7 | 158 | 0.004 |
| Abdominal obesity | 6.3 | 1.4 | 27.8 | 0.014 | 9.8 | 1.3 | 75 | 0.028 |
| Atherogenic dyslipemia† | 1.9 | 0.7 | 5.5 | 0.234 | 2.3 | 0.8 | 6.9 | 0.134 |
| Cholesterol remnants | 1.02 | 0.99 | 1.04 | 0.238 | 1.01 | 0.98 | 1.04 | 0.446 |
| LDL cholesterol | 0.9997 | 0.99 | 1.01 | 0.959 | 0.996 | 0.98 | 1.01 | 0.583 |
| HDL cholesterol < 40/50 | 1.4 | 0.5 | 3.6 | 0.513 | 1.9 | 0.7 | 5.3 | 0.207 |
| Age | 0.96 | 0.9 | 1.003 | 0.066 | 0.97 | 0.9 | 1.03 | 0.374 |
| Female sex | 0.3 | 0.1 | 0.9 | 0.035 | 0.7 | 0.3 | 1.9 | 0.475 |

†Atherogenic dyslipidemia is defined by triglyceride > 150 mg/dL and HDL-C < 40 mg/dL in men and < 50 mg/dL in women.

SUPPLEMENTARY TABLE 5. Changes in mean metabolic parameters from baseline according to progression/regression in liver fibrosis during follow-up.

1. Weight

|  | **Overall** | | | | | **NAFLD** | | | | **Dysglycemia** | | | |
| --- | --- | --- | --- | --- | --- | --- | --- | --- | --- | --- | --- | --- | --- |
| 8.0 kPa | Mean | SD | Range | | | Mean | SD | Range | | Mean | SD | Range | |
| Regression | -2.5 | 7.5 | -23 | 19 | -3.1 | | 7.8 | -23 | 19 | -3.6 | 7.2 | -23 | 7 |
| Maintained | 0.7 | 5.4 | -47 | 39 | -0.5 | | 5.9 | -47 | 20 | -0.5 | 5.4 | -24 | 29 |
| Progression | 3.2 | 4.9 | -10 | 11 | 3.0 | | 5.1 | -10 | 11 | 2.2 | 5.4 | -10 | 11 |
|  |  |  |  |  |  | |  |  |  |  |  |  |  |
|  |  |  |  |  |  | |  |  |  |  |  |  |  |
|  | **Overall** | | | | | **NAFLD** | | | | **Dysglycemia** | | | |
| 9.2 kPa | Mean | SD | Range | | | Mean | SD | Range | | Mean | SD | Range | |
| Regression | -5.3 | 7.5 | -23 | 7 | -5.8 | | 7.7 | -23 | 7 | -4.7 | 8.0 | -23 | 7 |
| Maintained | 0.7 | 5.4 | -47 | 39 | -0.4 | | 6.0 | -47 | 20 | -0.5 | 5.4 | -24 | 29 |
| Progression | 3.5 | 5.2 | -10 | 11 | 3.1 | | 5.2 | -10 | 11 | 2.6 | 5.8 | -10 | 11 |

Data are in kg

1. Waist circumference

|  | **Overall** | | | | | **NAFLD** | | | | **Dysglycemia** | | | |
| --- | --- | --- | --- | --- | --- | --- | --- | --- | --- | --- | --- | --- | --- |
| 8.0 kPa | Mean | SD | Range | | | Mean | SD | Range | | Mean | SD | Range | |
| Regression | -1.0 | 7.3 | -25 | 15 | -1.3 | | 7.7 | -25 | 15 | -2.1 | 7.3 | -25 | 7 |
| Maintained | 1.7 | 7.3 | -29 | 57 | 0.5 | | 7.3 | -29 | 57 | 1.5 | 7.6 | -29 | 57 |
| Progression | 2.6 | 6.3 | -22 | 12 | 2.2 | | 6.6 | -22 | 12 | 1.7 | 7.9 | -22 | 12 |
|  |  |  |  |  |  | |  |  |  |  |  |  |  |
|  |  |  |  |  |  | |  |  |  |  |  |  |  |
|  | **Overall** | | | | | **NAFLD** | | | | **Dysglycemia** | | | |
| 9.2 kPa | Mean | SD | Range | | | Mean | SD | Range | | Mean | SD | Range | |
| Regression | -3.4 | 7.7 | -25 | 6 | -3.6 | | 7.8 | -25 | 6 | -2.8 | 8.2 | -25 | 6 |
| Maintained | 1.7 | 7.3 | -29 | 57 | 0.5 | | 7.2 | -29 | 57 | 1.3 | 7.6 | -29 | 57 |
| Progression | 4.3 | 5.0 | -6 | 12 | 4.2 | | 5.2 | -6 | 12 | 3.4 | 5.9 | -6 | 12 |

Data are in cm.

1. Glucose

|  | **Overall** | | | | | **NAFLD** | | | | **Dysglycemia** | | | |
| --- | --- | --- | --- | --- | --- | --- | --- | --- | --- | --- | --- | --- | --- |
| 8.0 kPa | Mean | SD | Range | | | Mean | SD | Range | | Mean | SD | Range | |
| Regression | -6.5 | 40.4 | -198 | 68 | -6.5 | | 43.4 | -198 | 68 | -16.4 | 51.2 | -198 | 50 |
| Maintained | 0.9 | 18.1 | -208 | 126 | 0.9 | | 24.3 | -160 | 126 | -2.9 | 32.3 | -208 | 126 |
| Progression | 13.4 | 42.6 | -65 | 184 | 17.7 | | 45.9 | -65 | 184 | 17.3 | 52.0 | -65 | 184 |
|  |  |  |  |  |  | |  |  |  |  |  |  |  |
|  |  |  |  |  |  | |  |  |  |  |  |  |  |
|  | **Overall** | | | | | **NAFLD** | | | | **Dysglycemia** | | | |
| 9.2 kPa | Mean | SD | Range | | | Mean | SD | Range | | Mean | SD | Range | |
| Regression | -12.9 | 55.2 | -198 | 68 | -12.8 | | 57.8 | -198 | 68 | -25.6 | 65.8 | -198 | 50 |
| Maintained | 0.8 | 18.0 | -208 | 126 | 0.7 | | 23.8 | -160 | 126 | -3.2 | 31.7 | -208 | 126 |
| Progression | 21.2 | 47.8 | -65 | 184 | 25.5 | | 48.9 | -65 | 184 | 25.8 | 54.0 | -65 | 184 |

Data are in mg/dl.

1. HbA1c

|  | **Overall** | | | | | **NAFLD** | | | | **Dysglycemia** | | | |
| --- | --- | --- | --- | --- | --- | --- | --- | --- | --- | --- | --- | --- | --- |
| 8.0 kPa | Mean | SD | Range | | | Mean | SD | Range | | Mean | SD | Range | |
| Regression | -0.22 | 0.88 | -4.0 | 0.9 | -0.27 | | 0.93 | -4.0 | 0.9 | -0.39 | 1.13 | -4.0 | 0.9 |
| Maintained | 0.04 | 0.46 | -4.1 | 4.2 | 0.06 | | 0.62 | -4.1 | 4.2 | 0.04 | 0.79 | -4.1 | 4.2 |
| Progression | 0.39 | 0.99 | -1.5 | 3.3 | 0.48 | | 1.07 | -1.5 | 3.3 | 0.38 | 1.08 | -1.5 | 3.3 |
|  |  |  |  |  |  | |  |  |  |  |  |  |  |
|  |  |  |  |  |  | |  |  |  |  |  |  |  |
|  | **Overall** | | | | | **NAFLD** | | | | **Dysglycemia** | | | |
| 9.2 kPa | Mean | SD | Range | | | Mean | SD | Range | | Mean | SD | Range | |
| Regression | -0.43 | 1.25 | -4.0 | 0.9 | -0.49 | | 1.30 | -4.0 | 0.9 | -0.64 | 1.47 | -4.0 | 0.9 |
| Maintained | 0.04 | 0.46 | -4.1 | 4.2 | 0.05 | | 0.61 | -4.1 | 4.2 | 0.03 | 0.78 | -4.1 | 4.2 |
| Progression | 0.63 | 1.03 | -0.5 | 3.3 | 0.69 | | 1.06 | -0.5 | 3.3 | 0.62 | 1.02 | -0.5 | 3.3 |

Data are in %.
